# lncRNA–disease association prediction method based on the nearest neighbor matrix completion model

**DOI:** 10.1038/s41598-022-25730-0

**Published:** 2022-12-15

**Authors:** Xiao-xin Du, Yan Liu, Bo Wang, Jian-fei Zhang

**Affiliations:** College of Computer and Control, Qiqihar University, Qiqihar, 161006 China

**Keywords:** Cancer, Computational biology and bioinformatics, Diseases, Oncology

## Abstract

State-of-the-art medical studies proved that long noncoding ribonucleic acids (lncRNAs) are closely related to various diseases. However, their large-scale detection in biological experiments is problematic and expensive. To aid screening and improve the efficiency of biological experiments, this study introduced a prediction model based on the nearest neighbor concept for lncRNA–disease association prediction. We used a new similarity algorithm in the model that fused potential associations. The experimental validation of the proposed algorithm proved its superiority over the available Cosine, Pearson, and Jaccard similarity algorithms. Satisfactory results in the comparative leave-one-out cross-validation test (with AUC = 0.96) confirmed its excellent predictive performance. Finally, the proposed model’s reliability was confirmed by performing predictions using a new dataset, yielding AUC = 0.92.

## Introduction

Long noncoding ribonucleic acids (lncRNAs)^1^ exceeding 200 nucleotides in length have been erroneously treated as negligible (noise) RNAs^2,3^. However, recently they were found to be involved in dosage compensation effects, regulation of cell differentiation, epigenetic regulation and cell differentiation, cell proliferation, and cell cycle regulation such as apoptosis, and play essential roles in various life activities^4^. In particular, researchers have revealed that lncRNAs, such as H19, HOTAIR, and MALAT1, are very closely related to human diseases. While these lncRNAs are associated with the production of numerous human cancers, only a few have been experimentally related to particular human diseases. Therefore, analyzing available lncRNA–disease associations and predicting potential human lncRNA–disease associations have become essential tasks of bioinformatics, which would benefit the understanding of complex human disease mechanisms at the lncRNA level, disease biomarker detection and disease diagnosis, treatment, prognosis, and prevention^5,6^. Researchers have proposed numerous methods, which can be generally divided into two categories: the first is based on machine learning methods, and the other is based on network methods. Huang et al.^7^ also analyzed the latest relevant models to provide referenceable research directions for future ones from different perspectives.

General computational models use common machine learning-based computational models to process the data. For example, Chen et al.^8^ used semisupervised learning to predict potential associations between lncRNAs and diseases and proposed the first lncRNA–disease association prediction model (LRLSLDA). The semisupervised approach could be implemented without any negative disease-lncRNA association, which was the main advantage of this method. It opened new horizons for scholars to study lncRNAs, providing a reference model of lncRNA–disease research. To optimize the model, Huang et al.^9^ refined the calculation of disease similarity based on the framework of LRLSLDA. They improved the prediction results further and proposed a new method, ILNCSIM, which preserved the general hierarchical structure information of the disease DAG and determined the disease similarity calculation based on an edge-based approach. Finally, the prediction performance was improved to some extent. As the study continued, researchers discovered that the available data on lncRNA diseases were insufficient. Some researchers have developed methods that rely on information other than known lncRNA–disease associations to address this limitation. For example, Liu et al.^10^ proposed a method to identify potential lncRNA disease associations based on consistent gene-disease associations and gene-lncRNA co-expression relationships. This first computational method did not rely on known lncRNA–disease associations. Alternatively, Lan et al. constructed a web server for lncRNA–disease association prediction without relying on known associations^11^. A graph regression-based unified framework (GRUF) was proposed by Shi et al.^12^**,** which differed from most existing methods in that it could deal with lncRNAs without known disease associations and with any lncRNAs without diseases with known associations. Similar characteristics were intrinsic to the KATZLDA model proposed by Chen^13^. Eventually, some researchers attempted to use the concept of *K*-nearest neighbors to perform the analysis based on the above. For example, Xie et al.^14^ proposed a similarity kernel fusion (SKF-LDA) method to predict lncRNA disease associations. It exploited two different similarities, namely functional and semantic ones, through a novel fusion approach. Neighbor-based constraints on a refined similarity matrix constructed the fusion step. Additionally, applying the *K*-nearest neighbor concept, Cui et al.^15^ proposed a nearest profile-based association model approach called BLM-NPAI, which was constructed based on the original BLM that took into account NP information. Therefore, it could predict new lncRNAs using the nearest neighbor of each lncRNA and the disease without any association and new diseases. However, when introducing nearest neighbors, some noise might also be introduced to interfere with the prediction. Several researchers used probabilistic models. Probability-based modeling refers to treating a machine learning algorithm's input and output data as random variables and then modeling the problem from a probabilistic viewpoint. For example, Li et al.^16^ proposed a new weighted correlation method to construct a reliable lncRNA gene co-tabulation network based on logistic function transformation. They used statistical methods to screen out the lncRNAs associated with gastric cancer, which results were used in the subsequent experiments. Some other researchers applied Bayesian strategies to analyze the problem. Thus, Yu et al.^17^ introduced an NBCLDA approach based on a plain Bayesian classifier to predict potential lncRNA–disease associations. This method involved constructing a global network by integrating three heterogeneous networks (lncRNA- and miRNA-disease association networks and miRNA‒lncRNA interaction network). Besides, the gene-lncRNA interaction network, gene-disease association network, and gene-miRNA interaction network were added to the tripartite network forming a quadratic global network. The advantage of NBCLDA was that it could predict possible associations between lncRNAs and diseases contained in known association sets and potential associations whose elements were absent in the available datasets. Yu et al.^18^ also proposed a new CFNBC method based on a plain Bayesian classifier to predict lncRNA–disease associations. This method also constructed the original lncRNA–miRNA-disease tripartite network by integrating known miRNA–lncRNA associations, miRNA-disease associations, and lncRNA–disease associations. The novelty of CFNBC was the introduction of an item-based collaborative filtering algorithm and a plain Bayesian classifier, which ensured that CFNBC could effectively predict potential lncRNA–disease associations without relying exclusively on known miRNA-disease associations.

On the other hand, network-based learning methods were implemented by designing network models with various methods, such as random wandering (RW), heterogeneous networks, and propagation algorithms. Several researchers implemented random wandering on networks, attempting to reveal potential associations between lncRNAs and diseases with the RW approach. For example, Sun et al.^19^ proposed a global network-based RWRLNCD approach to predict potential disease associations of lncRNAs. Known lncRNA–disease association and similarity networks were used to construct functional similarity networks of lncRNAs. Subsequently, RW was reactivated in the functional similarity network of lncRNAs to predict potential lncRNA–disease associations. Chen et al.^20^ improved the conventional RW restart and proposed an improved random wandering restart method for lncRNA–disease association prediction (IRWRLDA). Likewise, LNCPRICNET^21^ used a multilevel composite network that integrated genes, lncRNAs, and their associated data to prioritize disease-associated candidate lncRNAs by restarting the random walk (RWR) algorithm. Hu et al.^22^ proposed a new algorithm for predicting lncRNA disease associations based on BiwalkLDA with double random walks. Similarly, Wen et al.^23^ proposed using Laplace normalization and double random walk on heterogeneous networks for predicting lncRNA disease associations. It differed from the previous model’s construction by normalizing the lncRNA similarity matrix and disease similarity matrix using the Laplace method as transpose before constructing the lncRNA similarity network and disease similarity network matrices and then associating them with existing lncRNA diseases. The weighted average of RW on both networks was used as a predictor of lncRNA disease correlation. The final double RW was applied to the heterogeneous network to predict the potential association between lncRNAs and diseases. Gradually, researchers switched to heterogeneous networks. Network-based lncRNA–disease association prediction featured a learning network of lncRNA–disease associations using known associations. The heterogeneous networks contained richer semantic and structural information than common ones. For example, Ganegoda et al.^24^ developed a computational model of the KRWRH network, a heterogeneous network consisting of a disease-disease similarity network, a lincRNA-lincRNA similarity network, and a known lincRNA-disease association network. Based on these methods, LNCPRED^25^ used network-based data to predict new ncRNA-disease associations to improve the accuracy of ncRNA and disease predictions. Considering the law that biological entities with the same or similar behavior are often related, Zhang et al.^26^ proposed a new computational framework, LNCRDNETFLOW, to infer potential lncRNA disease associations. It was based on a generic network prioritization model^27^, which implied constructing three similarity/interaction networks (lncRNA, disease, and protein) and three different mutual association networks (lncRNA disease, disease protein, and lncRNA protein). The global network was then built by integrating heterogeneous networks of interactions or similarities between biological entities (diseases, proteins, lncRNAs) and prioritizing the nodes. A flow propagation algorithm considering network topology information was also proposed to calculate global distances and predict potential lncRNA–disease associations. Numerous studies have proved that miRNAs usually interacted with lncRNAs and jointly participated in disease development. Therefore, miRNAs can be used as a bridge to study lncRNAs and diseases. Meanwhile, some scholars tried to clarify the relationship between lncRNAs and miRNAs. A model called LMI-INGI was proposed by Zhang et al.^28^. They applied a semisupervised interactome network-based approach to explore and forecast the latent interaction between lncRNAs and miRNAs. Chen et al.^29^ introduced a hypergeometric distribution model for lncRNA–disease association inference by integrating miRNA-disease associations and lncRNA‒miRNA interactions. Zhang et al.^30^ presented a network distance analysis model (NDALMA) for lncRNA–miRNA association prediction. The prediction scores were derived by integrating similarity networks to analyze network distances. Similarly, Zhang et al.^31^ applied a semisupervised interactome network-based approach to explore and forecast the latent interaction between lncRNAs and miRNAs. They constructed graphs based on the similarity of lncRNAs-miRNAs and combined known interactions to calculate scores as predicted outcomes. Chen et al.^32^ elaborated a new computational model named “Neighborhood Constraint Matrix Completion for MiRNA–Disease Association Prediction” (NCMCMDA) to predict potential miRNA–disease associations. They innovatively integrated neighborhood constraint with matrix completion, providing a novel idea of utilizing similarity information to assist the prediction. Immediately afterward, Chen et al.^33^ developed a deep-belief network model for miRNA-disease association prediction (DBNMDA). Compared with the previous supervised models, DBNMDA innovatively utilized the information of all miRNA-disease pairs during the pretraining process. This reduced the impact of too few known associations on prediction accuracy to some extent. Fan et al.^34^ developed the IDHI-MIRW approach to predict potential lncRNA disease associations based on a large-scale lncRNA disease heterogeneity network. It involved three lncRNA-related data types (lncRNA expression profiles, lncRNA‒miRNA interactions, and lncRNA protein interactions) in forming three lncRNA similarity networks and three disease-related information (disease semantic similarity, disease miRNA association, and disease gene association) to form three disease similarity networks. The lncRNA topological similarity networks, disease topological similarity networks, and known lncRNA–disease bipartite graphs were combined to construct large-scale lncRNA disease heterogeneity networks. Then, the candidate lncRNAs for each query disease were prioritized using the RWRH algorithm. Alternatively, Sudipto et al.^35^ proposed ranking LncRNAs using network diffusion (LION). This network diffusion approach integrated lncRNA, protein–protein, and disease protein networks to prioritize important lncRNAs in diseases. First, they constructed a network of lncRNA proteins, proteins-protein, and disease proteins in a multilevel complex network (triple network). Next, they applied a random walk network diffusion algorithm. The proximity of lncRNAs to disease genes was measured based on the probability of connecting edges. Which lncRNA was associated with a given disease was determined based on the probability of accessibility in the heterogeneous network. A model called the DWLMI was introduced by Yang et al.^36^. They inferred the potential associations between lncRNAs and miRNAs by representing them as vectors via a lncRNA‒miRNA-disease-protein-drug graph. There are some other models to associate protein and miRNA data with building heterogeneous networks. For example, Zhou et al.^37^ introduced a novel computational method to predict lncRNA–disease associations. They integrated associations between microRNAs (miRNAs), lncRNAs, proteins, drugs, and diseases to construct a heterogeneous network and then trained predictive models with a rotating forest classifier. Alternatively, Yuan et al.^38^ developed a machine-learning approach named LGDLDA. They computed similarity matrices from multivariate data and then integrated the neighborhood information in the similarity matrix using nonlinear feature learning of neural networks. Finally, LGDLDA ranked candidate lncRNA–disease pairs and then selected potential disease-related lncRNAs. Similarly, Li et al.^39^ proposed an approach called DF-MDA. They constructed a heterogeneous network by integrating various known associations between miRNAs, diseases, proteins, long noncoding RNAs (lncRNAs), and drugs. They then classified miRNA-disease associations using a random forest classifier. Noteworthy is that cyclic RNAs and metabolites were found to be somehow inextricably linked to the generation of disease and could serve as complementary data for lncRNA–disease studies^40,41^.

This paper proposes a method for prediction by the matrix completion technique inspired by recommender systems. Matrix completion is a common strategy in recommendation systems. Collaborative filtering algorithms in recommendation systems are a matrix completion technique. There are two kinds of collaborative filtering algorithms: a memory-based collaborative filtering algorithm and a model-based collaborative filtering algorithm. Memory-based collaborative filtering mainly uses heuristics to make recommendations by using similarity as weights and nearest neighbors to fill in missing values for user-item matrices to predict user needs and make recommendations, including both user-based and item-based algorithms; model-based collaborative filtering such as hidden semantic model and matrix factorization is based on matrix complementation theory, which is the extension of compressed perception theory from A low-rank and sparse matrix can be restored to a complete matrix with high accuracy^42^. The user-item matrix in recommendation systems is primarily a low-rank and sparse matrix. This theory can restore an entire matrix with no missing values to simulate a score for the user and recommend high-scoring items. Since the implicit semantic model and matrix decomposition have low explanatory power and high time cost in the face of large-scale data, this paper proposes a two-layer multi-weighted nearest-neighbor prediction model using a method similar to memory-based collaborative filtering, where neighbors are assigned weights to reassign values to the target matrix. The target matrix is an adjacency matrix consisting of lncRNAs and diseases. Relevant lncRNAs and diseases are marked as one at the corresponding position in the matrix, while unknown relationships are marked as 0. The size of the reassigned matrix elements represents the degree of correlation between lncRNAs and diseases. A higher value indicates a higher correlation. We can filter out the lncRNAs with high correlation for researchers to conduct biological experiments, thus narrowing the scope of experiments to improve research efficiency, which is a guide for biomedical experiments. This model provides a reliable solution to the prediction problem of sparse data. When the data are extremely sparse, the accuracy of the similarity calculation is improved by correlating correlated data, thus enabling the model to achieve satisfactory prediction results. This paper's available data in the lncRNA–disease dataset were less than 0.1%. The AUC value of the fivefold cross-validation experiment reached more than 0.94 after the correlation-related dataset assisted the similarity calculation. The code and experimental data are publicly available at https://github.com/nrgz/DMWNN-data.

## Materials

This study integrated three different datasets: the lncRNA–disease relationship dataset, the miRNA‒lncRNA relationship dataset, and the miRNA-disease relationship dataset. These were taken from the HMDD, starBase v2.0, and MNDR v2.0 databases, respectively. After comparing and removing duplicate values, we extracted 1089 lncRNA data, 373 disease data, and 246 miRNA data, as shown in Table 1.

**Table 1:** Experimental data statistics.

Data	lncRNAs	miRNAs	Disease	Interactions
lncRNA–miRNA	1089	246	–	9089
miRNA–disease	–	246	373	4704
lncRNA–disease	1089	–	373	407

The lncRNA–disease relationship, miRNA‒lncRNA relationship, and miRNA-disease relationship were used to construct the adjacency matrices **LD**, **ML**, and **MD**. lncRNA–disease relationships were extracted by merging and removing duplicate values from **LD**, **ML**, and **MD** to form the target matrix **Y.** In **Y,** if the lncRNA was associated with the disease, the corresponding position element was set to 1. If the lncRNA was not associated with the disease, the corresponding position element was set to 0. **Y** was a matrix of 1089 rows and 373 columns, containing 407 nonzero entries. Detailed data are in the referenced supplementary information (Supplementary informations 1, 2 and 3).

## Method

### Similarity calculation method with potential association attributes

In previous similarity calculations, {0, 0, 0, 0} and {1, 1, 1, 1} in the adjacency matrix were often defined as unrelated, where 1 and 0 represented proven and unproven associations, respectively. However, zero terms have the potential to be transformed into unity. Based on this assumption, a similarity calculation method incorporating the potential association property was proposed. The data initially considered irrelevant were given weights to participate in the calculation. The specific algorithm is described by Eq. 1:

1 $$sim\left(\mathbf{X},\mathbf{Y}\right)=\frac{\lambda {\Vert \mathbf{X}\times \mathbf{Y}\Vert }_{2}+\left(1-\lambda \right){\Vert \mathbf{X}-\mathbf{Y}\Vert }_{2}}{{\Vert {\varvec{\Gamma}}\Vert }_{2}}$$

where λ is the weight parameter, **Γ** is a vector with the same dimensions as **X** and **Y,** and each element is 1. **X** and **Y** are vectors with the same dimensions and elements consisting of 0 and 1. $\mathbf{X}\times \mathbf{Y}$ is the exterior product between vectors **X** and **Y**. The result is a vector.

#### LncRNA similarity

The **LMD** matrix with lncRNA as row miRNA and disease as the column was constructed with **LD**, **ML**, and **MD** matrices, and the similarity matrix ${\mathbf{S}}^{l}$ was calculated and built according to Eq. 2.

2 $${{\mathbf{S}}^{l}}_{i,j}=\frac{\lambda {\Vert {{\mathbf{L}\mathbf{M}\mathbf{D}}_{i}\times }_{ }{\mathbf{L}\mathbf{M}\mathbf{D}}_{j}\Vert }_{2}+\left(1-{\varvec{\lambda}}\right){\Vert {{\mathbf{L}\mathbf{M}\mathbf{D}}_{i}-}_{ }{\mathbf{L}\mathbf{M}\mathbf{D}}_{j}\Vert }_{2}}{{\Vert {{\varvec{\Gamma}}}_{l}\Vert }_{2}}$$

where ${\mathbf{L}\mathbf{M}\mathbf{D}}_{i}$ and ${\mathbf{L}\mathbf{M}\mathbf{D}}_{j}$ denote the i-th and j-th rows of the matrix $\mathbf{L}\mathbf{M}\mathbf{D}$, respectively, ${{\varvec{\Gamma}}}_{l}$ is a vector with the same dimension as ${\mathbf{L}\mathbf{M}\mathbf{D}}_{i}$ and all elements are 1, and $\lambda$ is the weight parameter.

#### Disease similarity

The **DML** matrix with lncRNA as row miRNA and disease as the column was constructed with **LD**, **ML**, and **MD** matrices. The similarity matrix ${\mathbf{S}}^{d}$ was calculated and built according to Eq. (3).

3 $${{\mathbf{S}}^{d}}_{i,j}=\frac{{\varvec{\lambda}}{\Vert {\mathbf{D}\mathbf{M}\mathbf{L}}_{i}\times {\mathbf{D}\mathbf{M}\mathbf{L}}_{j}\Vert }_{2}+\left(1- \lambda \right){\Vert {\mathbf{D}\mathbf{M}\mathbf{L}}_{i}-{\mathbf{D}\mathbf{M}\mathbf{L}}_{j}\Vert }_{2}}{{\Vert {{\varvec{\Gamma}}}_{d}\Vert }_{2}}$$

where ${\mathbf{D}\mathbf{M}\mathbf{L}}_{i}$ and ${\mathbf{D}\mathbf{M}\mathbf{L}}_{j}$ denote the i-th and j-th rows of the matrix **DML**, respectively ${{\varvec{\Gamma}}}_{d}$ is a vector with the same dimension as ${\mathbf{D}\mathbf{M}\mathbf{L}}_{i}$ and all elements are 1, and $\lambda$ is the weight parameter.

### Double multi-weighted nearest neighbor model

**Figure 1:**
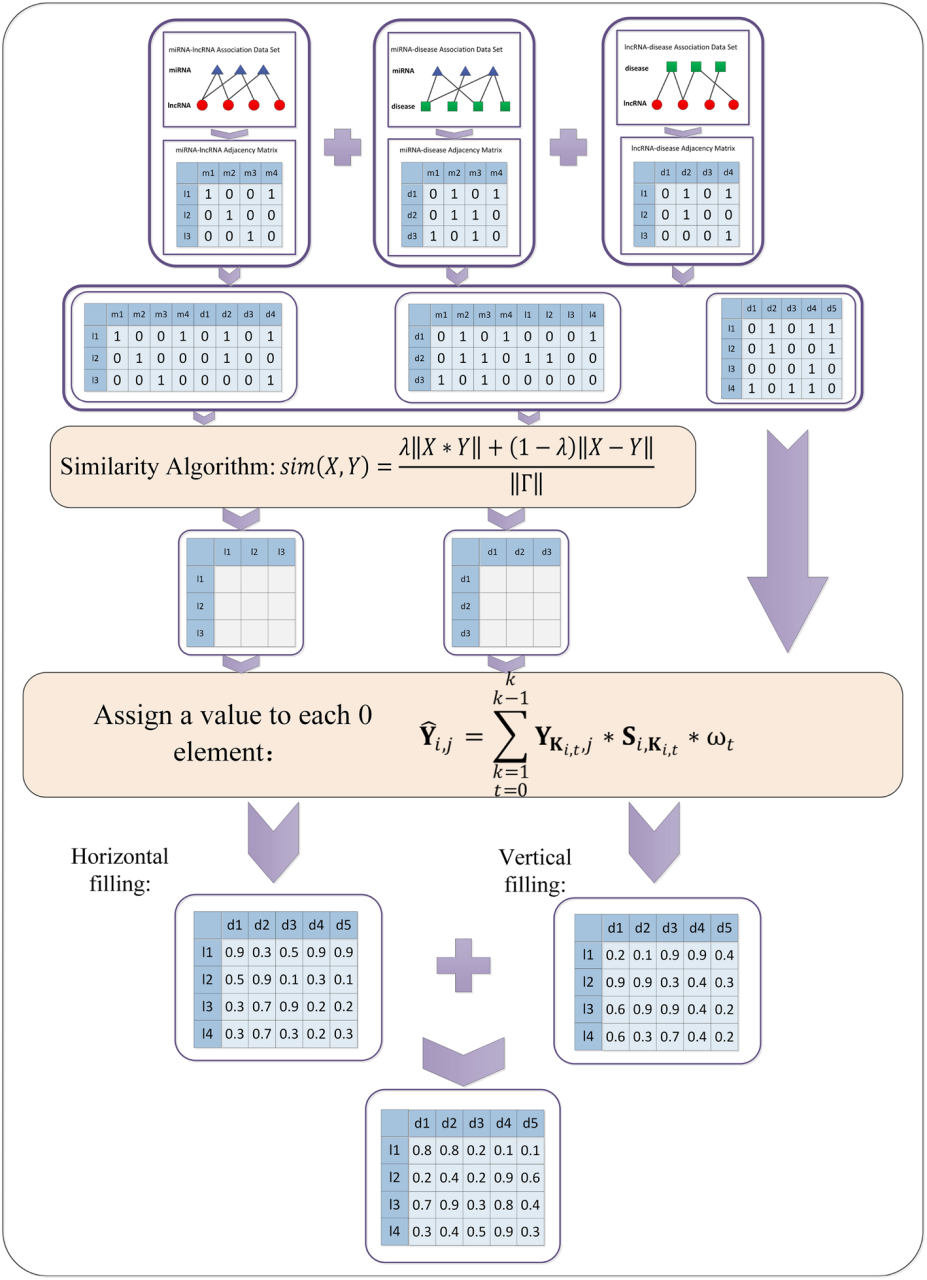
The single-layer model flow chart.

The double multi-weighted nearest neighbor model (DMWNN) was inspired by the memory-based collaborative filtering algorithm, unlike the recommendation algorithm, as a potential association prediction model between lncRNAs and diseases. It does not need to distinguish whether the main body is a user vector or an item vector but only needs to mine the association between lncRNAs and diseases as much as possible. Therefore, the DMWNN model can fill new values for the 0 items in the matrix from row and column vector perspectives and fuse the two filling results as the final. Figure 1 illustrates the construction process of the single-layer model.

The steps of model construction were as follows:

**Step 1.** Construct the index matrix* k* based on the correlation. Taking ${\mathbf{S}}^{l}$ as an example, put the first* k* values with larger values in row *i* of the matrix ${\mathbf{S}}^{l}$ into row *i* of matrix ${\mathbf{K}}^{l}$ in the order from highest to lowest.

4 $${{\mathbf{K}}^{l}}_{i,j}= {{{\varvec{S}}}_{l}^{\mathrm{^{\prime}}}}_{i,j} ;\left( j\in \left[0,\right.\left.k\right)\right)$$

where matrix ${\mathbf{S}}_{l\boldsymbol{ }}^{\mathbf{^{\prime}}}$ is the matrix obtained by sorting each row of the matrix ${\mathbf{S}}^{l}$ in descending order, and ${{{\varvec{S}}}_{l}^{\mathrm{^{\prime}}}}_{i,j}$ is the number of rows in the matrix ${\mathbf{S}}^{l}$ that rank *j* in similarity with the i*-*th row.

**Step 2.** Different weights are assigned to objects at different distances, with high weights for close objects and low weights for the opposite. This model uses a linearly decreasing weight assignment method, and the $t$-th close neighbor weight is:

5 $${\omega }_{t}=\frac{2*\left(k-t\right)}{k*\left(k+1\right)}$$

where* k* is the number of nearest neighbors, ω is the distance weight, and *t* is the ranking of the neighbors.

**Step 3.** The row vectors in the target matrix ${\mathbf{Y}}$ are processed according to Eq. (6).

6 $$ \widehat{{\mathbf{Y}}}_{{1_{i,j} }} = \left\{ {\begin{array}{*{20}l} {\sum\limits_{{\begin{array}{*{20}c} {k = 1} \\ {t = 0} \\ \end{array} }}^{{\begin{array}{*{20}c} k \\ {k - 1} \\ \end{array} }} {{\mathbf{Y}}_{{{\mathbf{K}}^{l}_{i,t} ,j}} {*}{\mathbf{S}}^{l}_{{i,{\mathbf{K}}^{l}_{i,t} }} {*}\omega_{t} ,} } \hfill & {if\quad {\mathbf{Y}}_{i,j} = 0} \hfill \\ {1,} \hfill & {else} \hfill \\ \end{array} } \right. $$

New values are filled for each row 0 entry to obtain the matrix ${\widehat{\mathbf{Y}}}_{1}$.

**Step 4.** The column vectors in the target matrix $\mathbf{Y}$ are processed according to Eq. (7).

7 $$ \widehat{{\mathbf{Y}}}_{{2_{i,j} }}^{T} = \left\{ {\begin{array}{*{20}l} {\sum\limits_{{\begin{array}{*{20}c} {k = 1} \\ {t = 0} \\ \end{array} }}^{{\begin{array}{*{20}c} k \\ {k - 1} \\ \end{array} }} {{\mathbf{Y}}^{{\text{T}}}_{{{\mathbf{K}}^{d}_{i,t,} j}} {*}{\mathbf{S}}^{d}_{{i,{\mathbf{K}}^{d}_{i,t} }} {*}\omega_{t} } } \hfill & {if\quad {\mathbf{Y}}^{{\text{T}}}_{i,j} = 0} \hfill \\ {1,} \hfill & {else} \hfill \\ \end{array} } \right. $$

New values are filled for the 0 entries in each column to obtain the matrix ${\widehat{\mathbf{Y}}}_{2}$.

**Step 5.** The matrix ${\widehat{\mathbf{Y}}}_{1}$ is fused with the matrix ${\widehat{\mathbf{Y}}}_{2}$ according to Eq. (8) to obtain the matrix ${\widehat{\mathbf{Y}}}_{0}$.

8 $$ {\hat{\mathbf{Y}}}_{{0_{i,j} }} = \eta_{1} {*}{\hat{\mathbf{Y}}}_{{1_{i,j} }} + \eta_{2} {*}{\hat{\mathbf{Y}}}_{{2_{i,j} }} $$

where ${\eta }_{1}$ and ${\eta }_{2}$ are the weight parameters. In this model, ${\eta }_{1}$ and ${\eta }_{2}$ are taken as 0.5.

**Step 6.** The row vectors of the ${\widehat{\mathbf{Y}}}_{0}$ matrix are processed according to Eq. (9).

9 $$ {\hat{\mathbf{Y}}}_{{1_{i,j} }}^{\user2{^{\prime}}} = \left\{ {\begin{array}{*{20}l} {\sum\limits_{{\begin{array}{*{20}c} {k = 1} \\ {t = 0} \\ \end{array} }}^{{\begin{array}{*{20}c} k \\ {k - 1} \\ \end{array} }} {{\hat{\mathbf{Y}}}_{{0_{{{\mathbf{K}}^{l}_{i,t} ,j}} }} {*}{\mathbf{S}}^{l}_{{i,{\mathbf{K}}^{l}_{i,t} }} {*}\omega_{t} ,} } \hfill & {if\quad {\hat{\mathbf{Y}}}_{{0_{i,j} }} = 0} \hfill \\ {1,} \hfill & {else} \hfill \\ \end{array} } \right. $$

New values are filled for the 0 entries in each row to obtain the matrix ${{\widehat{\mathbf{Y}}}_{1}}^{\mathrm{^{\prime}}}$.

**Step 7.** The column vectors of the ${\widehat{\mathbf{Y}}}_{0}$ matrix are processed according to Eq. (10).

10 $$ {\hat{\mathbf{Y}}}_{{2_{i,j} }}^{{\user2{^{\prime}T}}} = \left\{ {\begin{array}{*{20}l} {\sum\limits_{{\begin{array}{*{20}c} {k = 1} \\ {t = 0} \\ \end{array} }}^{{\begin{array}{*{20}c} k \\ {k - 1} \\ \end{array} }} {{\hat{\mathbf{Y}}}_{{0_{{{\mathbf{K}}^{d}_{i,t,} j}} }}^{{\text{T}}} {*}{\mathbf{S}}^{d}_{{i,{\mathbf{K}}^{d}_{i,t} }} {*}\omega_{t} ,} } \hfill & {if\quad {\hat{\mathbf{Y}}}_{{0_{i,j} }}^{T} = 0} \hfill \\ {1,} \hfill & {else} \hfill \\ \end{array} } \right. $$

The 0 entries in each column are filled with new values to obtain the matrix ${{\widehat{\mathbf{Y}}}_{2}}^{\mathbf{^{\prime}}}$.

**Step 8.** The matrix ${{\widehat{\mathbf{Y}}}_{1}}^{\mathbf{^{\prime}}}$ is fused with ${{\widehat{\mathbf{Y}}}_{2}}^{\mathrm{^{\prime}}}$ to obtain the final prediction matrix $\widehat{\mathbf{Y}}$ according to Eq. (11).

11 $$ {\hat{\mathbf{Y}}}_{i,j} = \eta_{1} \user2{*}{\hat{\mathbf{Y}}}_{{1_{i,j} }}^{\user2{^{\prime}}} + \eta_{2} \user2{*}{\hat{\mathbf{Y}}}_{{2_{i,j} }}^{\user2{^{\prime}}} $$

**Figure 2:**
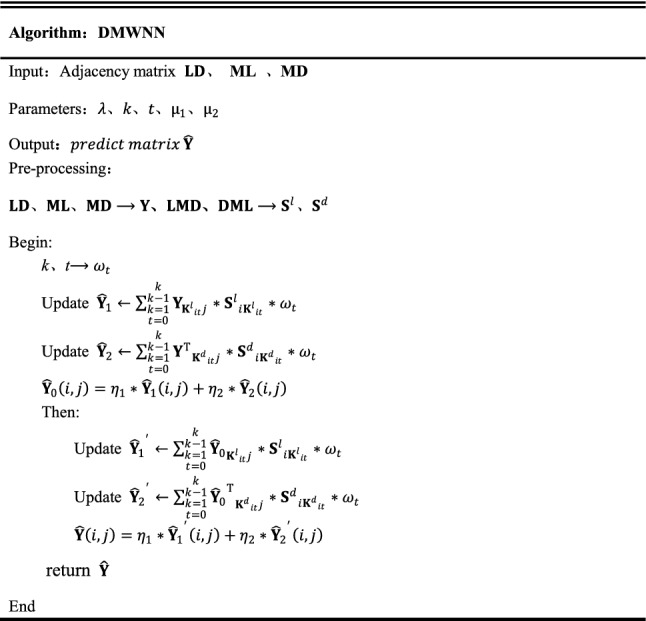
DMWNN model pseudo-code.

Figure 2 shows the pseudocode of the DMWNN model, illustrating the execution process of the algorithm.

## Results and discussion

### Cross-validation

Cross-validation is a standard method for model training when the amount of data is insufficient. Usually, model training requires data splitting into a training set, test set, and validation set. This implies that the training set has less data than the original data, and the validation set can contain only some initial data. The cross-validation method can use all the data for training and validation. For example, the fivefold cross-validation method can split the data into five parts, taking one as the validation set and the rest as the training set each time and repeating the experiment five times. Using the average performance of the five experiments as the model performance under the current parameters, one can also avoid the problem of overfitting. The final evaluation of the proposed method’s quality is the “area under the curve” (AUC) value^43^. It is usually defined as the area under the receiver operating characteristic (ROC) curve. The false positive rate (FPR, 1-specificity) represents the abscissa of the ROC curve. The true positive rate (TPR, sensitivity) is the ordinate of the ROC curve, and the calculation formulas for FPR and TPR are given in Eqs. (12) and (13), respectively:

12 $$FPR=\frac{FP}{TN+FP}$$

13 $$TPR=\frac{TP}{TP+FN}$$

where TP and FP are the numbers of positive samples with true and false classifications, respectively. Similarly, TN and FN are the numbers of negative samples with true and false classifications, respectively.

Previous studies show that the AUC values are between 0 and 1. The method is feasible only if the AUC ranges between 0.5 and 1^44^.

### Similarity metric evaluation

The Cosine, Pearson, and Jaccard similarity correlation coefficients were selected for comparison in this study's performance evaluation experiments. As the accuracy of similarity algorithms couldn’t be obtained by direct comparison, several similarity algorithms were used separately in prediction models to reflect the merits of similarity algorithms by the performance of their respective models. Since the DMWNN two-layer model based on the Cosine similarity failed to fully meet the requirement of assigning values to all zero terms, the three-layer nearest neighbor model was used to evaluate the performance. The fivefold cross-validation method was chosen to represent the model's predictive performance by the average performance obtained five times.

**Figure 3:**
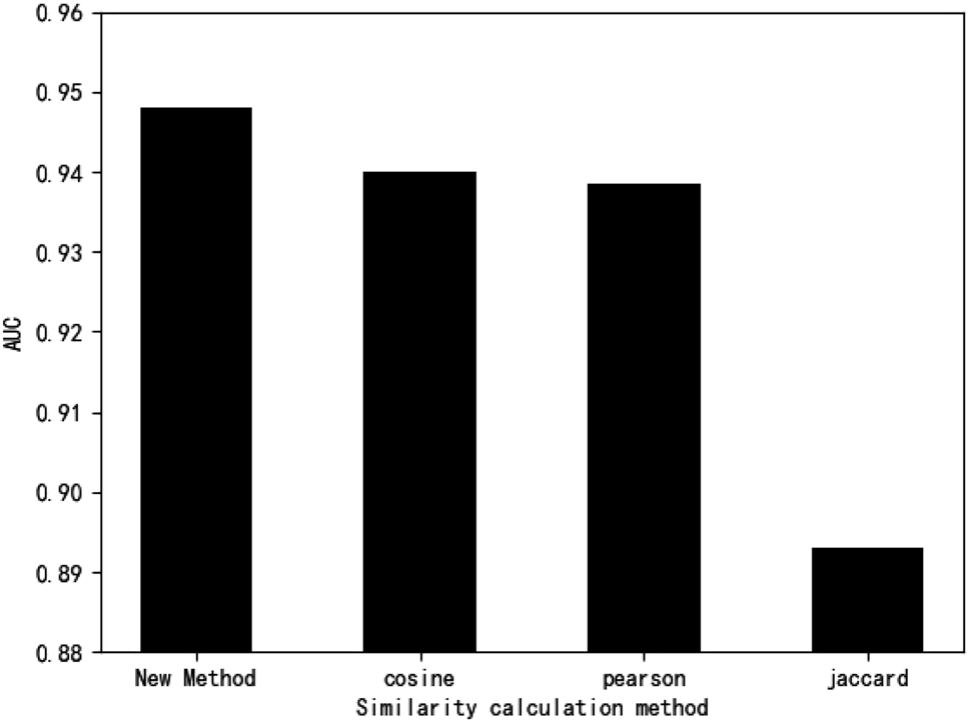
Performance evaluation.

In the fivefold cross-validation experiments, we manually adjusted the parameters many times based on the results of each experiment to obtain the best performance for each model. The experiments yielded that the improved calculation method, Cosine, Pearson, and Jaccard similarity correlation coefficients reached their optimal performance at* k* = {217, 268, 276, 323} with AUC values of {0.9477, 0.9399, 0.9385, 0.8930}, respectively. From Fig. 3, it can be seen that the improved similarity calculation method outperformed all other methods under study.

### Double multi-weighted nearest neighbor model

#### Performance evaluation of the double multi-weighted nearest neighbor model

This trial used the lncRNA–disease relationship dataset, miRNA‒lncRNA relationship dataset, and miRNA-disease relationship dataset from the HMDD, starBase v2.0, and MNDR v2.0 databases, respectively, containing 1089 lncRNAs, 246 miRNAs, and 373 diseases.

**Figure 4:**
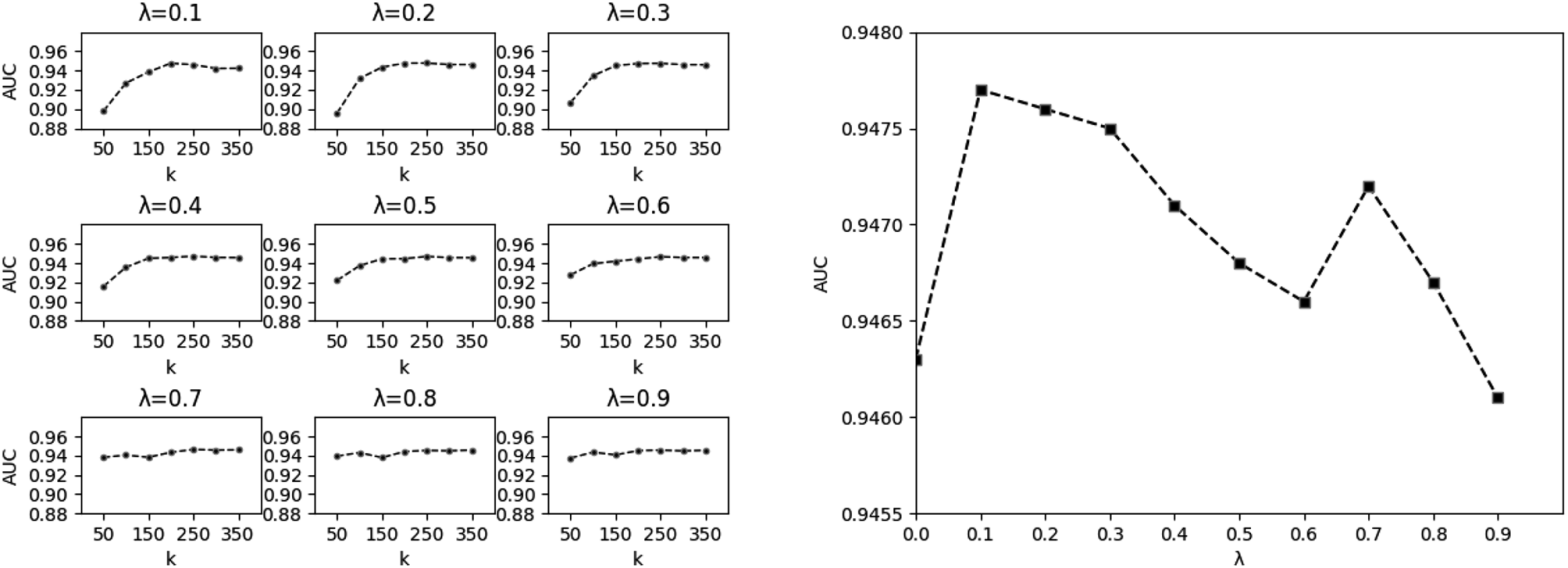
Performance fluctuations of the model at different values of* k* and λ (Left) and trend of model optimal performance with λ (Right).

First, we used fivefold cross-validation to select the optimal parameters for the model, and the weight parameter λ was chosen from {0, 0.1, 0.2, 0.3, 0.4, 0.5, 0.6, 0.7, 0.8, 0.9}. The performance variation at different parameters is shown in Fig. 4.

We continuously adjusted the parameters through fivefold cross-validation experiments according to the above performance trends so that the models corresponding to different λ achieved the best performance. The respective performance reached the optimum when* k* was taken {300, 217, 220, 260, 259, 263, 262, 373, 373, 372} by the experimental verification (see Table 2). The trend of performance fluctuation is shown in Fig. 4. The highest AUC value of 0.9477 was reached at the weight parameter λ = 0.1, providing the model’s best performance.

**Table 2:** Performance comparison of DMWNN model with different parameters.

Similarity parameter λ	0	0.1	0.2	0.3	0.4	0.5	0.6	0.7	0.8	0.9
k	300	217	220	260	259	263	262	373	373	372
AUC	0.9463	0.9477	0.9476	0.9475	0.9471	0.9468	0.9466	0.9472	0.9467	0.9461

To more comprehensively evaluate this model, we used a broader range of evaluation criteria, including accuracy (Acc.), sensitivity (Sen.), specificity (Spec.), precision (Prec.), and the Matthews correlation coefficient (MCC). The prediction performance is listed in Table 3. The average Acc., Sen., Spec., Prec., MCC, and AUC values were 91.64, 92.01, 91.65, 1.21, 9.80 and 93.82%, respectively, when using the proposed method to predict lncRNA–disease associations. The standard deviations of these values were 2.11, 3.31, 2.12, 0.48, 2.16 and 1.91%, respectively. Although the model had low scores in Pre and MCC, on balance, this model was a reliable predictor. At the same time, the lower standard deviation of these standards implied that the proposed model was robust and stable.

**Table 3:** Fivefold cross-validation results of our method.

Fold	Acc. (%)	Sen. (%)	Spec. (%)	Prec. (%)	MCC (%)	AUC (%)
1	93.60	87.78	93.61	1.51	11.04	91.47
2	90.58	91.03	90.58	0.93	8.66	92.09
3	88.40	97.83	88.39	0.48	6.42	96.82
4	91.39	90.72	91.39	1.25	10.10	94.49
5	94.25	92.71	94.25	1.89	12.78	94.23
Average	91.64 ± 2.11	92.01 ± 3.31	91.65 ± 2.12	1.21 ± 0.48	9.80 ± 2.16	93.82 ± 1.91

#### Multilayer model comparison

Since the target matrix *Y* was too sparse, even if the number of nearest neighbors* k* was set to the maximum, the single-layer model would fail to achieve the purpose of assigning values to all 0 items. Therefore, a multilayer model was adopted to superimpose the processing.The more stacked layers, the smaller the minimum* k* value to meet the requirement. This implies that the maximum* k* value that can be selected for the next stacking also becomes smaller. If* k* is no less than 3, the model will detect that there are no more zero items in the matrix Y after five stacking processes, and the sixth process will be avoided. Experimentally, the minimum* k* value of the 5-layer model was 3, and the maximum* k* value used to continue the stacked model execution was 2. At* k* equal to 1 or 2, the stacking had to contain more than five layers to meet the assignment requirements. However, the stacking of more than five layers was not considered to ensure that the model would have less complexity and higher generalization ability.

**Figure 5:**
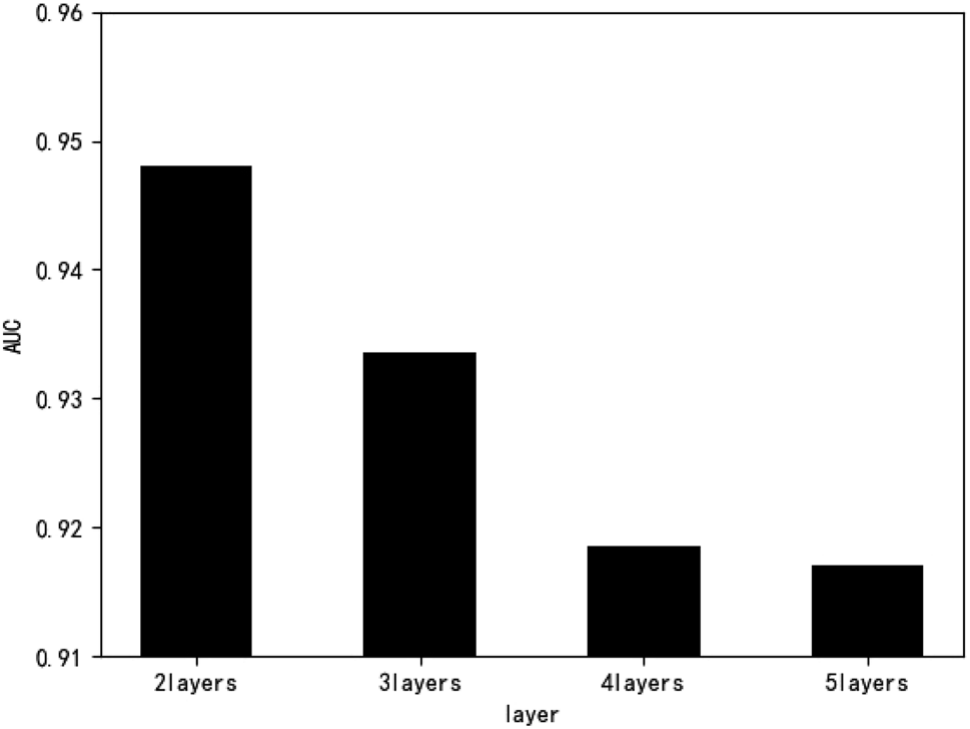
Performance comparison of different layer models.

The same fivefold cross-validation method was used, and the average performance obtained five times was used to represent the model's prediction performance. The parameters were manually adjusted to achieve the best performance for each multilayer model based on the results of each experiment. The two-layer model was experimentally verified to obtain the optimal performance. That of each model is described in Table 4. Figure 5 shows that the two-layer model outperformed all other models, so it was chosen as the final prediction model. The prediction performance deteriorated with the number of layers, probably because each layer's prediction was an iteration of the previous layer's prediction result, resulting in increasingly unrealistic forecasts.

**Table 4:** Performance of models with different number of layers.

Layers	2	3	4	5
Similarity parameter λ	0.1	0.9	0.8	0.9
k	217	32	4	3
AUC	0.9477	0.9336	0.9185	0.9171

#### Performance comparison with previous models

**Figure 6:**
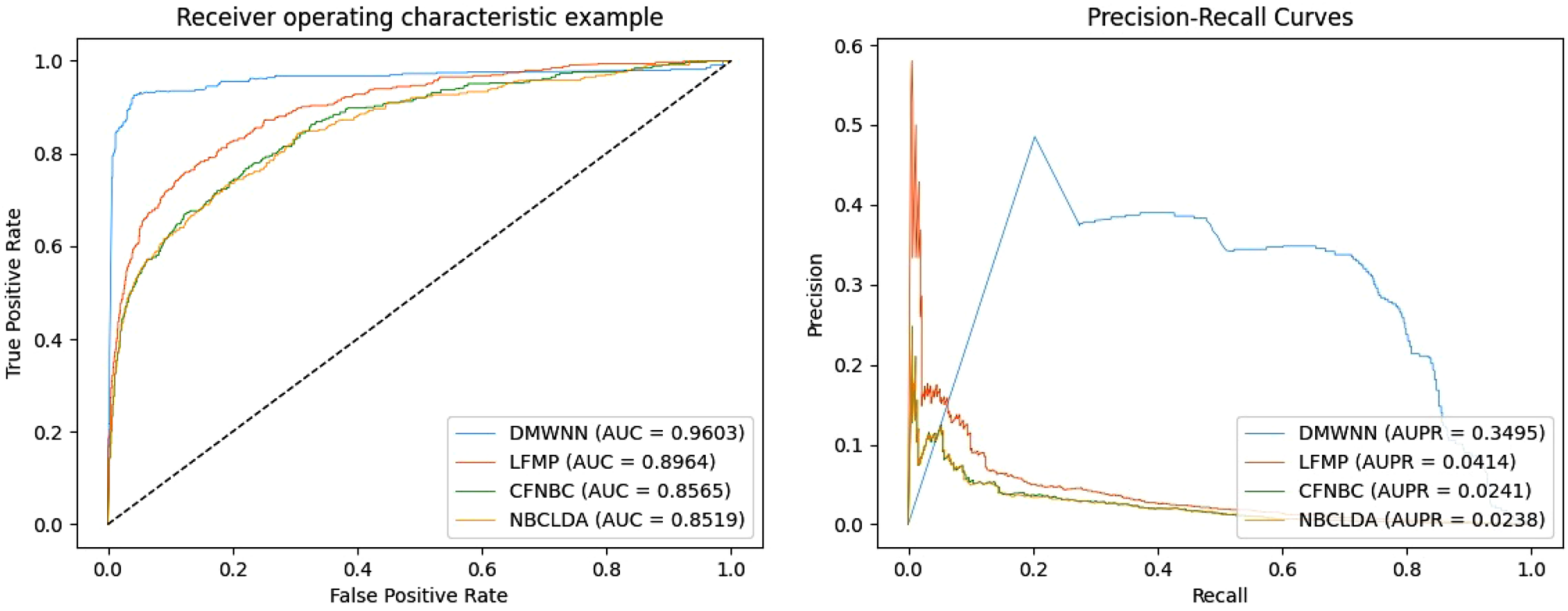
The performance of DMWNN in terms of ROC curves (Left) and PR curves (Right) based on 407 known lncRNA–disease associations under the LOOCV frameworks.

The AUC values of {0.9603, 0.8694, 0.8565, 0.8519} were obtained by testing this model, as well as the LFMP^45^, CFNBC^18^, and NBCLDA^17^ models, using the leave-one-out cross-validation under the same dataset. The AUC values of the DMWNN model proposed in this paper significantly exceeded those of the other models, demonstrating the best prediction performance. The ROC and AUPR comparison charts based on LOOCV are plotted in Fig. 6.

To better examine the model's predictive performance, we used a new dataset for comparison with other models. The results are shown in Table 5. The data were collected from Lnc2Cancer, LncRNADisease, GeneRIF, HMDD (v2.0), and starBase. In total, they contained 240 lncRNAs, 495 miRNAs, and 412 diseases. It can be seen that the AUC of DMWN reached 0.923, exceeding those of other models in the tested data. In particular, this AUC value exceeded that of SIMCLDA^46^ by 24%, MFLDA^47^ by 47%, LDAP^11^ by 7%, and Ping’s method^48^ by 6%. Moreover, DMWNN achieved an AUPR of 0.340, outperforming all other techniques involved in the comparison. Specifically, it outperformed SIMCLDA by 258%, MFLDA by 415%, LDAP by 105%, and Ping’s method by 55%, proving its excellent prediction ability.

**Table 5:** The AUCs and AUPRs of different prediction models.

Algorithm	AUC	AUPR
SIMCLDA	0.746	0.095
MFLDA	0.626	0.066
LDAP	0.863	0.166
Ping’s method	0.871	0.219
DMWNN	0.923	0.340

#### Case study

We selected four common cancers (namely, stomach neoplasm, lung neoplasm, colorectal neoplasm, and glioma) to analyze the actual prediction performance of the proposed model. By processing the adjacency matrix of lncRNA–disease using the DMWNN model, the scores of lncRNAs in the columns of several cancers were ranked in the final prediction matrix, and the top twenty lncRNAs were selected for validation. This paper tested the prediction results using literature and database validations through the PubMed index and LncRNADisease database.

**Figure 7:**
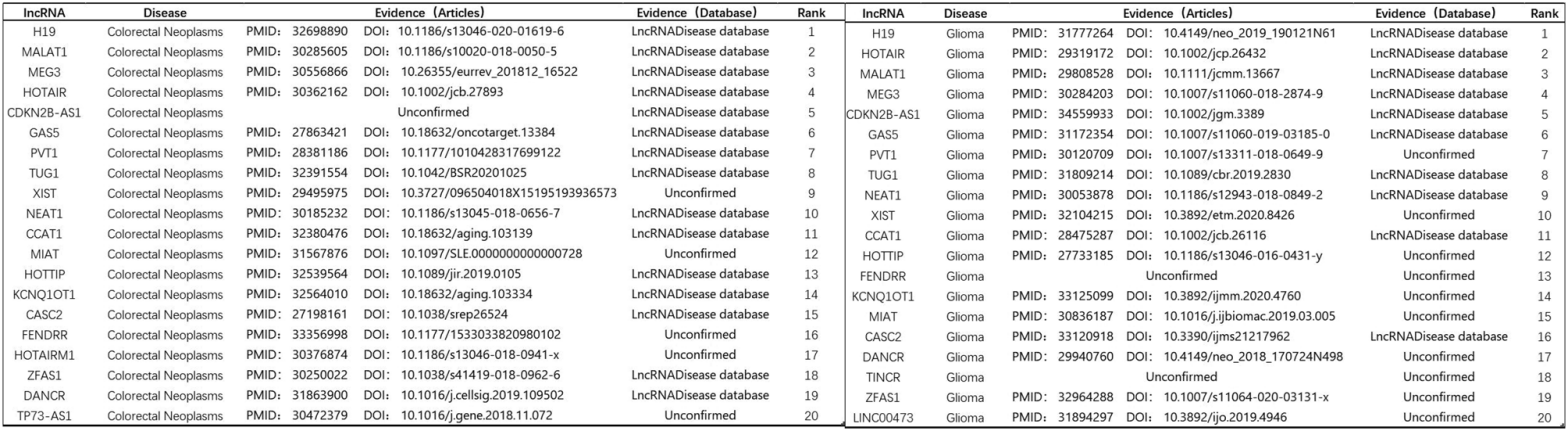
Validation results of lncRNAs predicted to be associated with colorectal neoplasm (Left) and glioma (Right).

**Figure 8:**
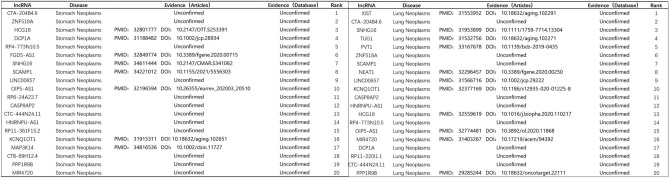
Validation results of lncRNAs predicted to be associated with stomach neoplasm (Left) and lung neoplasm (Right).

After examination, 19 of the 20 lncRNAs screened to predict association with colorectal tumors were validated, while 18 of the 20 lncRNAs screened to predict association with glioma were validated, as shown in Fig. 7. In the case of gastric and lung cancers, nearly half of the potential associations were successfully predicted by the latest literature validation despite the absence of relevant data in the database. The prediction results are shown in Fig. 8. The performed case analysis strongly indicates that the DMWNN model proposed in this paper has high prediction accuracy.

## Conclusions and model limitations

Recent research on long noncoding ribonucleic acids (lncRNAs) revealed their involvement in numerous human life activities and a key role in many pathological processes. While many biological experiments have explored the relationship between lncRNAs and diseases, it is still necessary to develop effective predictive models to assist biological experiments and improve experimental efficiency. This study adopted a simple and effective two-layer nearest neighbor model based on a similarity algorithm incorporating potential associations, which was suitable for the data obtained by constructing the adjacency matrix. Unlike other algorithms, it assigned weights to data initially judged to be unrelated and then participated in calculating similarity. This similarity algorithm was experimentally verified to outperform several similar algorithms, being the core of the proposed two-level nearest neighbor model. It screened the neighbors, based on the degree of similarity, as a crucial component of the prediction score. The other three components making up the score were the distance and distance weights between the neighbors. The multilayer model was designed to predict unknown data adequately. Since too many layers would bias the prediction data, it was experimentally verified that two layers provided the optimal model’s performance. The difference in performance produced by different datasets was evident in the comparison experiments. The first comparison experiment introduced miRNA in the similarity calculation, thus improving the similarity calculation accuracy. The results proved that the proposed model provided more accurate predictions when the amount of data was sufficient.

While the prediction model heavily relies on the similarity algorithm, its similarity calculation's accuracy also depends on the amount of data. Therefore, the proposed model is extremely sensitive to the data, and the prediction results may vary significantly from one dataset to another. Moreover, similarity calculation requires data with a high correlation, and the closer the correlation, the more accurate the similarity calculation. However, the lncRNAs or target diseases usually have less relevant data, deteriorating the correlation's prediction efficiency. In the follow-up study, we envisage combining miRNAs and proteins. Since lncRNAs generally interact with miRNAs and proteins to participate in various human life activities, the degree of their association is relatively high, and these data can be correlated to improve the model performance. Finally, our similarity calculation method is not complete enough and can only predict whether lncRNA is related to disease, which is still a far shot from screening out lncRNAs that are truly involved in disease formation. Given that lncRNAs have become critical regulators of cancer pathways and biomarkers of various diseases, we also intend to design more reasonable similarity calculation methods from gene expression and survival data to improve the prediction accuracy and use the results in targeted cancer therapy.

## Supplementary Information

Supplementary Information 1.

Supplementary Information 2.

Supplementary Information 3.

## Data Availability

The datasets analyzed during the current study are available in HMDD (http://www.cuilab.cn/hmdd), starBase v2.0 (https://starbase.sysu.edu.cn/starbase2/) and MNDR v2.0 (http://www.rna-society.org/mndr/) databases.
